# Association between asymptomatic hyperuricemia and kidney stones

**DOI:** 10.1371/journal.pone.0349819

**Published:** 2026-05-26

**Authors:** Jun Ho Lee, Suzy Park, Nuri Oh, Dae Yeon Cho, Jae Yoon Kim

**Affiliations:** 1 Department of Urology, Nowon Eulji Medical Center, Eulji University School of Medicine, Seoul, Republic of Korea; 2 School of Medicine, UHW Main Building, Heath Park, Cardiff University, Cardiff, United Kingdom; 3 Department of Chemistry and Biology, Korea Science Academy of Korea Advanced Institute of Science and Technology, Busan, Republic of Korea; 4 Department of Urology, Sanggye Paik Hospital, Inje University College of Medicine, Seoul, Republic of Korea; China Medical University, TAIWAN

## Abstract

**Background:**

Nephrolithiasis is commonly observed in patients with gout; however, the role of asymptomatic hyperuricemia in stone formation remains unclear. This study evaluated the association between serum uric acid levels and nephrolithiasis in a large health screening population.

**Methods:**

We conducted a cross-sectional analysis of 31,198 Korean adults who underwent health checkups between 2010 and 2020. Clinical parameters included anthropometric measures, blood pressure, serum uric acid, renal and hepatic function markers, lipid profiles, glycemic indices, inflammatory biomarkers, serum calcium, and vitamin D3 levels. Nephrolithiasis was defined as the presence of at least one renal stone ≥5 mm detected by ultrasonography and/or kidney–ureter–bladder radiography. Hierarchical logistic regression models (unadjusted, age- and BMI-adjusted, and fully adjusted) were used to assess associations separately in men and women.

**Results:**

Median serum uric acid concentrations were 6.0 mg/dL in men and 4.4 mg/dL in women. In men, higher serum uric acid levels were associated with nephrolithiasis across hierarchical models. In fully adjusted analyses, serum uric acid remained significantly associated with stone prevalence, and men in the highest uric acid quartile (≥6.9 mg/dL) had higher odds compared with those in the lowest quartile (adjusted OR: 3.546; 95% CI: 1.240–10.144). No statistically significant association was observed in women.

**Conclusions:**

Higher serum uric acid levels were associated with nephrolithiasis in men but not in women in this cross-sectional analysis. These findings contribute to the epidemiologic understanding of sex-specific differences in stone risk; prospective studies are needed to clarify causality and potential clinical implications.

## Introduction

Nephrolithiasis, or kidney stone disease, is a prevalent urological condition that affects approximately 10–15% of the global population during their lifetime [1]. Its incidence has steadily increased over the past several decades, consistent with increases in obesity, diabetes, metabolic syndrome, and other chronic non-communicable diseases [2,3]. Kidney stones are more common in men than in women [4], and their recurrence rate is substantial, often necessitating repeated imaging, interventions, and long-term management. The socioeconomic burden associated with nephrolithiasis is significant, including direct healthcare expenditures as well as indirect losses in productivity.

The pathogenesis of kidney stones is multifactorial, involving the supersaturation of urine with stone-forming constituents (such as calcium, oxalate, and uric acid), impaired excretion of inhibitory compounds (e.g., citrate), and alterations in urinary pH [5]. Hyperuricemia (elevated serum uric acid concentration) has been shown to promote uric acid stone formation under acidic urinary conditions in experimental rodent models. In these studies, uric acid was used as a crystalline nidus, facilitating calcium oxalate deposition and stone growth [6,7]. Furthermore, uric acid induces renal tubular oxidative stress and inflammation, altering the renal microenvironment and promoting lithogenesis [8,9]. These findings suggest potential mechanisms contributing to stone pathogenesis in humans.

Although the relationship between gout and nephrolithiasis is well established [10–12], the clinical implications of asymptomatic hyperuricemia in developing nephrolithiasis remain poorly understood. While asymptomatic hyperuricemia is not itself an indication for pharmacologic treatment, it is clinically relevant because it has been associated with various conditions such as hypertension, chronic kidney disease, and metabolic syndrome [13–15]. Given the public health importance of kidney stones and the widespread prevalence of asymptomatic hyperuricemia, clarifying their relationship would enhance our understanding of the pathological mechanisms underlying urolithiasis and aid in the development of preventive and therapeutic strategies. Therefore, we conducted this study to evaluate the association between asymptomatic hyperuricemia and nephrolithiasis by leveraging a large sample size and comprehensive clinical data.

## Methods

### Study population

**Fig 1:**
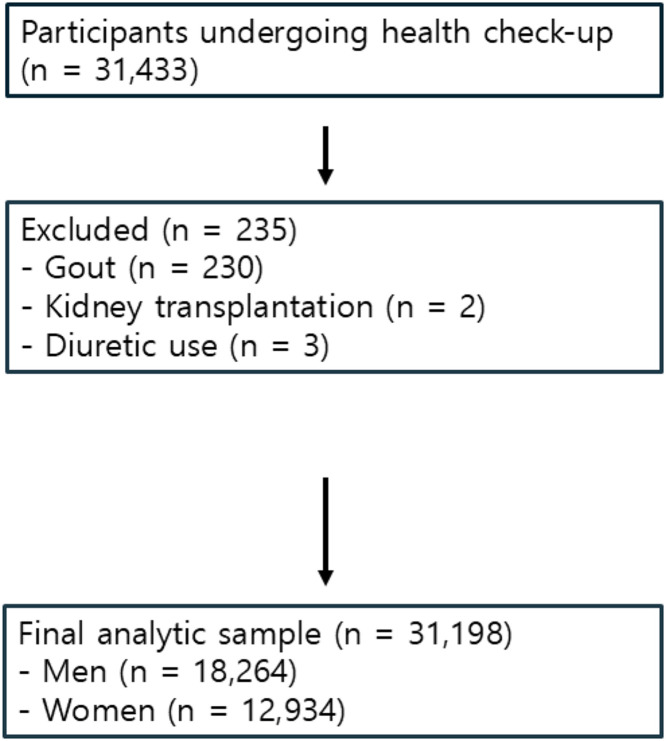
Participant Flow Diagram.

This retrospective study included adult participants aged ≥20 years who underwent standardized health screenings at an academic hospital in Seoul, South Korea, from January 2010 to December 2020. Data were obtained from individuals who voluntarily participated in comprehensive health checkups and were analyzed for research purposes (18/09/2025). Eligibility criteria included complete data on serum uric acid levels, abdominal ultrasonography, and kidney-ureter-bladder (KUB) radiography. Exclusion criteria included a history of gout (n = 230), congenital renal anomalies (e.g., polycystic kidney disease, dysgenesis, and hypoplasia), renal cancer or tumors, a history of kidney transplantation (n = 2), and the use of diuretics (n = 3). After applying the predefined exclusion criteria, 12,934 men and 18,264 women were included in the final analytic sample (Fig 1).

Asymptomatic hyperuricemia was defined as elevated serum uric acid measured at health screening in the absence of a prior diagnosis of gout, no history of urate-lowering therapy, and no documented symptomatic nephrolithiasis at baseline. Serum uric acid was analyzed as a continuous variable and additionally examined across sex-specific quartiles.

This study was approved by the Institutional Review Board of Inje University Sanggye Paik Hospital (IRB No. 2025-09-004). Due to the retrospective design and exclusive use of de-identified data, the requirement for informed consent was waived by the Institutional Review Board. The authors did not have access to information that could identify individual participants during or after data collection.

### Confounding variables

In previous studies, age [16], hypertension [17], diabetes mellitus (DM) [18], body mass index (BMI) [19], dyslipidemia [20], renal function [21], hs-CRP [22], HbA1c [23], vitamin D3 [24], and serum calcium [25] were reported as risk factors for urinary stones. Fatty liver disease has also been identified as a risk factor for nephrolithiasis; therefore, Aspartate Transaminase (AST) and Alanine Transaminase (ALT) levels were used as surrogate parameters for fatty liver disease [26]. We adjusted for these factors to elucidate the association between uric acid levels and nephrolithiasis.

### Clinical and laboratory data collection

Covariates were selected a priori based on clinical relevance and evidence from prior literature. Demographic and anthropometric variables (age, sex, and body mass index [BMI]), blood pressure measurements, and fasting laboratory parameters were collected.

Systolic and diastolic blood pressure, HbA1c, and lipid profiles were analyzed as continuous variables rather than as dichotomous diagnostic categories (e.g., hypertension, diabetes mellitus, or dyslipidemia). Sex was included as a categorical variable, whereas all other covariates were entered as continuous variables in the analyses.

Laboratory assessments included serum uric acid, creatinine, blood urea nitrogen (BUN), aspartate aminotransferase (AST), alanine aminotransferase (ALT), triglycerides, high-density lipoprotein (HDL) cholesterol, low-density lipoprotein (LDL) cholesterol, HbA1c, high-sensitivity C-reactive protein (hs-CRP), calcium, and 25-hydroxyvitamin D_3_. All blood samples were obtained between 7:00 AM and 9:00 AM after an overnight fast of at least 8 hours.

### Imaging and stone identification

To detect nephrolithiasis, we used both KUB radiography and transabdominal ultrasonography. KUB was performed before ultrasound to help localize potential stones, and both imaging modalities were interpreted by board-certified radiologists. Stones were suspected on KUB images when calcifications were observed along the anatomical course of the kidneys. Ultrasonography was performed using an iU22 system (Philips Medical Systems, Amsterdam, Netherlands) with B-mode imaging to identify bright echogenic foci with posterior acoustic shadowing, which is considered a diagnostic of renal stones. Echogenic foci <5 mm without shadowing were not classified as nephrolithiasis to focus on clinically significant stones, based on literature suggesting that asymptomatic stones ≥5 mm are more likely to become symptomatic over time [27,28]. Combining KUB with ultrasound improves sensitivity and specificity compared to ultrasound alone [29]. For statistical analysis, nephrolithiasis was coded as a binary variable (presence vs. absence).

### Statistical analysis

Descriptive statistics were used to compare the baseline characteristics between male and female participants. All analyses were conducted separately for men and women to account for known sex-specific differences in serum uric acid distribution and metabolism.

All analyses were performed separately for men and women to account for known sex-specific differences in serum uric acid distribution and metabolism (both as continuous and categorical variables by sex-specific quartiles).

Multivariable logistic regression models were constructed with a primary focus on clinically relevant confounders defined a priori based on existing literature and biological plausibility. These included age, body mass index, key metabolic parameters, and measures of renal function. Additional laboratory variables were included to assess the robustness of the association rather than to imply causal pathways. Because several metabolic biomarkers, particularly triglycerides and hs-CRP, showed markedly right-skewed distributions, additional analyses were conducted using sex-specific quartile categories for these variables as sensitivity analyses.

To evaluate the impact of confounding, hierarchical logistic regression analyses were performed. Three models were constructed: Model 1 (unadjusted), Model 2 (adjusted for age and BMI), and Model 3 (fully adjusted for metabolic, renal, hepatic, and inflammatory covariates). The fully adjusted model included systolic and diastolic blood pressure, lipid parameters, liver enzymes, renal function markers, glycemic indices, calcium, vitamin D3, and inflammatory markers. These hierarchical analyses were conducted separately for men and women.

Statistical significance was set as a two-tailed p-value of <0.05. All analyses were performed using R (R Foundation for Statistical Computing, Vienna, Austria). Analyses were conducted using a complete-case approach. Participants with missing data in key exposure, outcome, or covariate variables were excluded from the corresponding multivariable analyses. Although the proportion of missing data was low, complete-case analysis may introduce bias if missingness is not completely at random.

Analyses were conducted using a complete-case approach. Participants with missing data for the exposure, outcome, or covariates included in each model were excluded from the corresponding analyses. The proportion of missing data was low.

## Results

Detailed characteristics of the study population according to sex are summarized in Table 1. The final analytic sample included 31,198 individuals, including 12,934 women and 18,264 men. Nephrolithiasis was identified in 427 participants (1.4%) overall, with a higher prevalence in men (328/18,264, 1.8%) than in women (99/12,934, 0.8%). The median ages of male and female participants were 48.0 and 47.0 years, respectively. Men had significantly higher median serum uric acid levels (6.0 mg/dL) than women (4.4 mg/dL).

**Table 1:** Baseline characteristics of study participants by sex, including age, BMI, blood pressure, lipid profile, liver and kidney function tests, HbA1c, uric acid, hs-CRP, calcium, and vitamin D3.

	Male	Female
	(N = 18264)	(N = 12934)
Age (yr)	48.0 [41.0;55.0]	47.0 [39.0;55.0]
SBP (mmHg)	124.0 [116.0;132.0]	116.0 [107.0;127.0]
DBP (mmHg)	75.0 [69.0;82.0]	70.0 [64.0;77.0]
BMI (kg/m^2^)	22.3 [20.5;24.6]	24.8 [23.1;26.9]
TG (mg/dL)	126.0 [87.0;182.0]	84.0 [61.0;121.0]
LDL (mg/dL)	123.0 [100.0;146.0]	115.0 [94.0;138.0]
HDL (mg/dL)	47.0 [41.0;55.0]	59.0 [50.0;69.0]
AST (IU/L)	25.0 [21.0;32.0]	21.0 [18.0;26.0]
ALT (IU/L)	27.0 [20.0;39.0]	17.0 [13.0;24.0]
Cr	1.0 [0.9; 1.1]	0.8 [0.7; 0.8]
BUN	13.8 [11.8;16.2]	12.3 [10.3;14.7]
HbA1C	5.6 [5.3; 5.9]	5.5 [5.2; 5.7]
Uric acid	6.0 [5.2; 6.9]	4.4 [3.8; 5.1]
hs-CRP	0.1 [0.0; 0.1]	0.1 [0.0; 0.1]
Calcium	9.3 [9.1; 9.6]	9.2 [9.0; 9.5]
Vitamin D3	20.4 [15.2;26.7]	21.4 [14.6;29.4]

Values are presented as the median [interquartile range].

BMI = body mass index; SBP = systolic blood pressure; DBP = diastolic blood pressure; TG = triglyceride; LDL = low-density lipoprotein cholesterol; HDL = high-density lipoprotein cholesterol; AST = aspartate transaminase; ALT = alanine transaminase; BUN = blood urea nitrogen; Cr = creatinine; HbA1c=hemoglobin A1c; hs-CRP = high sensitive C-reactive.

Univariate logistic regression analysis in men revealed that older age, higher BUN, elevated serum uric acid, and higher vitamin D3 levels were significantly associated with kidney stones (Table 2).

**Table 2:** Unadjusted ORs for kidney stones in males and females.

	Male		Female	
	OR (95% CI)	p	OR (95% CI)	p
Age	1.029 (1.019-1.039)	.000	1.033 (1.017-1.050)	.000
SBP	1.008 (1.000-1.016)	.059	1.009 (.996-1.022)	.196
DBP	1.014 (1.003-1.025)	.012	1.016 (.997-1.036)	.105
BMI	1.023 (.988-1.058)	.197	1.021 (.964-1.083)	.473
TG	1.000 (.999-1.001)	.870	.998 (.995-1.002)	.408
LDL	.997 (.994-1.000)	.090	.994 (.988-1.000)	.055
HDL	.995 (.986-1.005)	.351	.979 (.965-.994)	.007
AST	1.001 (.998-1.003)	.573	1.000 (.999-1.001)	.932
ALT	1.001 (.996-1.005)	.809	1.000 (.999-1.002)	.529
Cr	.821 (.428-1.573)	.552	1.561 (.592-4.118)	.368
BUN	1.029 (1.008-1.051)	.008	1.001 (.947-1.058)	.962
HbA1C	1.076 (.929-1.246)	.329	1.005 (.688-1.468)	.978
Uric acid	1.125 (1.037-1.220)	.004	.891 (.726-1.095)	.274
hs-CRP	.709 (.399-1.261)	.242	1.000 (.463-2.162)	.999
Calcium	1.262 (.958-1.662)	.099	1.077 (.662-1.754)	.764
Vitamin D3	1.026 (1.003-1.048)	.025	.981 (.940-1.025)	.393

BMI = body mass index; SBP = systolic blood pressure; DBP = diastolic blood pressure; TG = triglyceride; LDL = low-density lipoprotein cholesterol; HDL = high-density lipoprotein cholesterol; AST = aspartate transaminase; ALT = alanine transaminase; BUN = blood urea nitrogen; Cr = creatinine; HbA1c=hemoglobin A1c; hs-CRP = high sensitive C-reactive protein.

After adjusting for confounders (Table 3), only age (adjusted odds ratio (OR), 1.053; 95% confidence interval (CI), 1.020–1.088) and serum uric acid levels (adjusted OR, 1.364; 95% CI, 1.068–1.743) remained independently associated with nephrolithiasis in men. Because triglycerides and hs-CRP exhibited right-skewed distributions, these variables were categorized into sex-specific quartiles and included in the fully adjusted models to reduce the influence of extreme values and improve model stability. (S1 Table).

**Table 3:** Multivariate logistic regression analysis of factors associated with nephrolithiasis in men and women.

	Male		Female	
	OR (95% CI)	p	OR (95% CI)	p
Age	**1.053 (1.020-1.088)**	**.002**	1.039 (.988-1.093)	.139
SBP	.990 (.956-1.026)	.596	1.016 (.967-1.067)	.533
DBP	1.003 (.960-1.048)	.888	1.004 (.937-1.076)	.906
BMI	1.059 (.964-1.163)	.233	.965 (.808-1.152)	.694
TG	.999 (.996-1.002)	.549	.987 (.973-1.001)	.069
LDL	.994 (.986-1.002)	.148	.995 (.982-1.009)	.506
HDL	.994 (.965-1.023)	.665	.979 (.943-1.017)	.279
AST	1.000 (.977-1.022)	.967	.967 (.914-1.024)	.250
ALT	1.006 (.986-1.026)	.582	1.039 (.993-1.088)	.099
Cr	.978 (.297-3.222)	.971	1.227 (.310-4.864)	.771
BUN	.993 (.918-1.075)	.868	1.004 (.879-1.147)	.948
HbA1C	1.044 (.705-1.547)	.828	1.294 (.648-2.585)	.465
Uric acid	**1.364(1.068–1.743)**	**.013**	.687 (.410–1.152)	.155
hs-CRP	.742 (.177-3.105)	.686	.017 (.000-27.600)	.279
Calcium	1.040 (.455-2.378)	.926	2.626 (.810-8.513)	.108
Vitamin D3	1.015 (.985-1.045)	.331	.965 (.919-1.012)	.143

Odds ratios represent the change in odds of nephrolithiasis per unit increase in each variable. The model was adjusted for age, BMI, blood pressure, lipid profile, liver enzymes, renal function markers, glycemic indices, calcium, vitamin D3, and inflammatory markers. Because triglycerides and hs-CRP exhibited right-skewed distributions, additional analyses using sex-specific quartiles were conducted as sensitivity analyses (S1 Table).

BMI = body mass index; SBP = systolic blood pressure; DBP = diastolic blood pressure; TG = triglyceride; LDL = low-density lipoprotein cholesterol; HDL = high-density lipoprotein cholesterol; AST = aspartate transaminase; ALT = alanine transaminase; BUN = blood urea nitrogen; Cr = creatinine; HbA1c=hemoglobin A1c; hs-CRP = high sensitive C-reactive protein.

In hierarchical logistic regression analyses (Table 4), the association between serum uric acid and nephrolithiasis in men remained significant across all stages of adjustment. In unadjusted analysis, each 1 mg/dL increase in serum uric acid was associated with higher odds of nephrolithiasis (OR 1.125, 95% CI 1.037–1.220). After adjustment for age and BMI, the association slightly strengthened (OR 1.184, 95% CI 1.087–1.288), and it remained significant in the fully adjusted model (OR 1.364, 95% CI 1.068–1.743). In contrast, no statistically significant association between serum uric acid and nephrolithiasis was observed in women at any stage of modeling. Although age and lower HDL cholesterol levels were associated with nephrolithiasis in unadjusted analyses (Table 2), these associations were attenuated and no longer statistically significant after multivariable adjustment (Table 3). These findings indicate a difference in observed associations by sex; however, formal interaction testing was not performed.

**Table 4:** Hierarchical logistic regression models for the association between serum uric acid and nephrolithiasis.

Model	Male OR (95% CI)	p	Female OR (95% CI)	p
Model 1 (Unadjusted)	1.125 (1.037–1.220)	.004	0.891 (0.726–1.095)	.274
Model 2 (Age + BMI adjusted)	1.184 (1.087–1.288)	<.001	0.875 (0.709–1.080)	.214
Model 3 (Fully adjusted)*	1.364 (1.068–1.743)	.013	0.687 (0.410–1.152)	.155

*Model 3 adjusted for age, BMI, blood pressure, lipid profile, liver enzymes, renal function markers, glycemic parameters, calcium, vitamin D3, and inflammatory markers (sex-specific quartile categories for triglycerides and hs-CRP).

Additionally, in men, serum uric acid levels categorized into quartiles demonstrated a graded association with nephrolithiasis. Both unadjusted and fully adjusted analyses showed higher odds of kidney stone presence with increasing uric acid levels. Compared with the first quartile, men in the highest quartile (≥6.9 mg/dL) had greater odds of nephrolithiasis, with an unadjusted OR of 1.427 (95% CI: 1.039–1.959) and an adjusted OR of 3.546 (95% CI: 1.240–10.144) (Table 5). In contrast, no statistically significant association between serum uric acid quartiles and nephrolithiasis was observed in women. Quartile stratification did not demonstrate a consistent trend in stone prevalence, and adjusted odds ratios were not statistically significant across categories (Table 5).

**Table 5:** Both absolute prevalence (number and percentage of cases) and adjusted odds ratios for nephrolithiasis across sex-specific serum uric acid quartiles. Reference group is the lowest quartile.

	Male				Female			
	Uric acid quartile (n), (uric acid level, mg/dL)				Uric acid quartile (n), (uric acid level, mg/dL)			
	Q1 (4648) (<5.2)	Q2 (4489) (≥5.2, <6.0)	Q3 (4740) (≥6.0, <6.9)	Q4 (4387) (≥6.9)	Q1 (3253) (<3.8)	Q2 (3232) (≥3.8, <4.4)	Q3 (3459) (≥4.4, <5.1)	Q4 (2990) (≥5.1)
Nephrolithiasis, number (%)	(68, 1.4%)	(77, 1.7%)	(92, 1.9%)	(91, 2.1%)	(30, 0.9%)	(27, 0.8%)	(19, 0.5%)	(23, 0.8%)
Unadjusted OR (95% CI)	1.00 (reference)	1.175 (.846-1.633)	1.333 (.972-1.829)	1.427 (1.039-1.959)	1.00 (reference)	.905 (.537-1.526)	.593 (.333-1.056)	.833 (.483-1.437)
p		.335	.075	.0.28*		.708	.076	.511
Adjusted OR (95% CI)^#^	1.00 (reference)	2.009 (.705-5.722)	2.372 (.830-6.783)	3.546 (1.240-10.14)	1.00 (reference)	1.038 (.301-3.576)	.277 (.0510-1.495)	.633 (.157-2.546)
p		.192	.107	.0.18*		.953	.136	.519

Values are presented as number (%).

Quartiles were defined separately for men and women.

Triglycerides and hs-CRP were categorized into sex-specific quartiles due to their right-skewed distributions.

#Adjusted odds ratios were derived from the primary multivariable model including age, body mass index, blood pressure, renal function, glycemic indices, and lipid parameters.

*p < 0.05; OR=odds ratio; CI = confidence interval, Q1 = 1^st^ quartile, Q2 = 2^nd^ quartile, Q3 = 3^rd^ quartile, Q4 = 4^th^ quartile.

**Fig 2:**
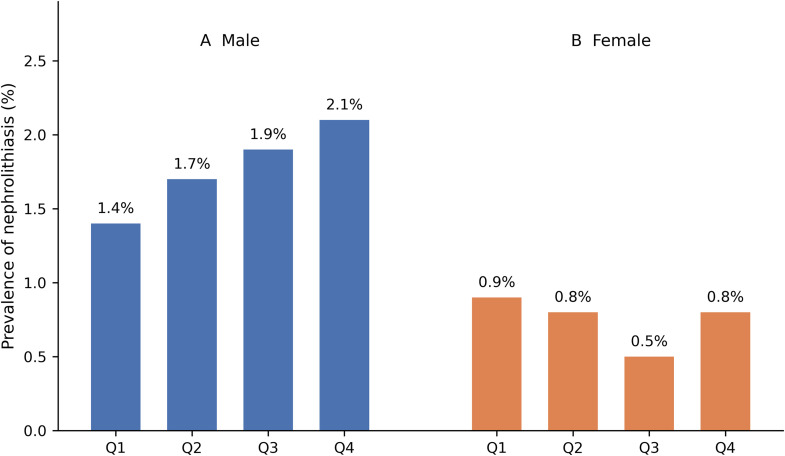
Absolute prevalence of nephrolithiasis according to sex-specific serum uric acid quartiles. Bars represent the proportion of participants with nephrolithiasis within each quartile. (A) Male participants (Q1: n = 4648, < 5.2 mg/dL; Q2: n = 4489, ≥ 5.2– < 6.0 mg/dL; Q3: n = 4740, ≥ 6.0– < 6.9 mg/dL; Q4: n = 4387, ≥ 6.9 mg/dL). (B) Female participants (Q1: n = 3253, < 3.8 mg/dL; Q2: n = 3232, ≥ 3.8– < 4.4 mg/dL; Q3: n = 3459, ≥ 4.4– < 5.1 mg/dL; Q4: n = 2990, ≥ 5.1 mg/dL).

The prevalence of nephrolithiasis was higher in men than in women. Across serum uric acid quartiles, an increasing prevalence of nephrolithiasis was observed in men, whereas no consistent trend was observed in women (Fig 2). Absolute prevalence and adjusted odds ratios across quartiles are presented in Table 5. These findings reflect sex-stratified associations and do not constitute a formal test of interaction between sex and serum uric acid. Estimates with wide confidence intervals, particularly in women, likely reflect limited statistical information and should be interpreted with caution.

## Discussion

Our findings indicate that higher serum uric acid levels were associated with the presence of nephrolithiasis in men, whereas no significant association was observed in women. This association remained directionally consistent across hierarchical models with progressive adjustment for demographic and metabolic variables, although the magnitude of effect should be interpreted cautiously given the inclusion of multiple correlated covariates.

A previous meta-analysis estimated a pooled OR of 1.77 for nephrolithiasis in patients with gout, a condition characterized by chronic hyperuricemia [10]. However, to date, only a few studies have investigated the correlation between asymptomatic hyperuricemia and kidney stones. In a cohort study conducted in South Korea, men with asymptomatic hyperuricemia had a significantly higher risk of developing kidney stones (based solely on ultrasound findings) than men with normal uric acid levels (adjusted OR = 1.72), whereas no such association was observed in women [30]. Another study conducted in China reported that asymptomatic hyperuricemia was associated with an increased OR of nephrolithiasis in both sexes (adjusted OR =1.464), with serum urate thresholds of 356 μmol/L in men and 265 μmol/L in women, beyond which kidney stone risk increased [31]. However, these previous studies did not adjust for key lithogenic risk factors, such as vitamin D and calcium levels, both of which were considered in our analysis, thereby strengthening the clinical relevance of our findings.

Mechanistically, uric acid may promote nephrolithiasis via several complementary pathways. At high concentrations, uric acid crystallizes in acidic urine, forming uric acid stones. Even in the absence of uric acid stones, monosodium urate crystals can be a nidus for calcium oxalate aggregation, which is the most common stone type. The inflammatory pathways activated by uric acid include NLRP3 inflammasome-mediated cytokine release and oxidative stress within renal tubular cells, leading to local epithelial injury, reduction of urinary stone inhibitors, and promotion of crystal adhesion and retention [32].

Urinary pH is a critical mediator of this process. Uric acid solubility decreases drastically in acidic urine (pH < 5.5), increasing the propensity for crystal formation. Individuals with similar serum uric acid levels may have different lithogenic risks, depending on their urine pH profiles [5,33]. Unfortunately, urine pH data were not available in our study cohort, limiting our ability to evaluate this mechanism directly. Nonetheless, the role of urinary acidification in uric acid stone formation is established and should be the focus of future investigation.

Sexual dimorphism in uric acid metabolism likely explains the lack of association observed in women. Estrogens upregulate uric acid excretion by enhancing the expression of urate transporters in the renal proximal tubules, thereby promoting more efficient clearance. Supporting this finding, hormone replacement therapy has been shown to reduce serum uric acid levels in postmenopausal women [34,35]. Additionally, women have a lower prevalence of metabolic syndrome, insulin resistance, and excess dietary purine intake, all of which may interact synergistically with hyperuricemia to promote lithogenesis [36].

In this study, given the comprehensive adjustment, the primary interpretation of the multivariable analysis focuses on the direction and consistency of the association rather than the exact magnitude of adjusted estimates.

In hierarchical modeling, the association between serum uric acid and nephrolithiasis in men was consistent across unadjusted, age/BMI-adjusted, and fully adjusted models. The effect estimate modestly increased after multivariable adjustment, suggesting that the observed association was not explained by measured metabolic or renal confounders. The consistency of effect estimates across hierarchical models further supports the robustness of the observed association in men. In contrast, no significant association was observed in women across any stage of adjustment.

Given the cross-sectional design, these findings should be interpreted as associations rather than evidence of causality. Although serum uric acid remained independently associated with nephrolithiasis in men after comprehensive adjustment, residual confounding cannot be entirely excluded. Furthermore, because multiple correlated metabolic variables were included in the fully adjusted model, overadjustment or multicollinearity may have influenced the precision of some estimates. Therefore, our primary interpretation focuses on the consistency and direction of the association rather than on the exact magnitude of adjusted odds ratios.

From a clinical perspective, our findings suggest that serum uric acid levels may be informative when considering nephrolithiasis risk, particularly in men. However, asymptomatic hyperuricemia is not currently an indication for pharmacologic intervention, and our results should be interpreted as associative rather than causal. Prospective studies will be required to determine whether modification of serum uric acid levels in selected individuals can reduce the risk of stone formation. As precision medicine advances, improved identification of individuals most likely to benefit from preventive strategies may help reduce the overall burden of nephrolithiasis.

This study had a few limitations. First, the cross-sectional design precluded the establishment of causality. Second, computed tomography was not used to confirm the presence of stones. Third, urinary biochemical parameters, such as pH, citrate, calcium, and oxalate levels were not measured. Fourth, we were unable to account for dietary intake or genetic predisposition, which may have influenced the risk of lithogenesis. Fifth, the study population consisted of individuals undergoing voluntary health check-ups at a single tertiary center. Such participants may be healthier, more health-conscious, or of higher socioeconomic status than the general population. Therefore, selection bias cannot be excluded, and the generalizability of these findings to broader community-based or multi-ethnic populations may be limited. In addition, because a complete-case analysis was used, the possibility of bias related to missing data cannot be entirely excluded, however, the proportion of missing data was low. Sixth, we did not formally test a sex-by–serum uric acid interaction; therefore, the observed differences between men and women should be interpreted as descriptive rather than confirmatory evidence of effect modification. Nevertheless, despite these limitations, the use of a large cohort and adjustment for several clinically relevant metabolic factors, including serum calcium and vitamin D levels, provides additional epidemiologic context for the observed association between asymptomatic hyperuricemia and nephrolithiasis.

## Conclusion

In summary, our study demonstrates a sex-specific association between serum uric acid levels and nephrolithiasis in a large health screening population. Higher uric acid levels were associated with stone prevalence in men but not in women. These findings contribute to the epidemiologic understanding of sex differences in stone risk; however, given the cross-sectional design, causal inference cannot be established. Prospective studies are required to determine whether serum uric acid plays a modifiable role in stone prevention.

## Supporting information

S1 Table Multivariable logistic regression analysis including sex-specific quartile categorization of triglycerides and hs-CRP. (DOCX)
