# Supplementary material for: Association between asymptomatic hyperuricemia and kidney stones
Source: PLoS One. 2026 May 26;21(5):e0349819. doi: 10.1371/journal.pone.0349819 (PMC13210304; doi:10.1371/journal.pone.0349819)
Supplement: S1 Table — (DOCX) [file pone.0349819.s001.docx]

**S1 Table.** Multivariable logistic regression analysis including sex-specific quartile categorization of triglycerides and hs-CRP

| **Variable** | **Category** | **Male OR (95% CI)** | **p-value** | **Female OR (95% CI)** | **p-value** |
| --- | --- | --- | --- | --- | --- |
| TG Q1 | Reference | 1.00 | – | 1.00 | – |
| TG Q2 | vs Q1 | .547(.210-1.423) | .216 | 5.821 (1.028-32.960) | .046 |
| TG Q3 | vs Q1 | 1.061 (.453-2.485) | .891 | 3.290 (.531-20.382) | .201 |
| TG Q4 | vs Q1 | .741 (.280-1.957) | .545 | 2.787 (.500-15.541) | .242 |
| hs-CRP Q1 | Reference | 1.00 | – | 1.00 | – |
| hs-CRP Q2 | vs Q1 | 1.042 (.409-2.657) | .931 | .513 (.144-1.825) | .303 |
| hs-CRP Q3 | vs Q1 | 1.134 (.460-2.794) | .785 | .178 (.034-.931) | .041 |
| hs-CRP Q4 | vs Q1 | .743 (.274-2.012) | .559 | .334 (.071-1.570) | .165 |

TG = triglyceride; hs-CRP=high sensitive C-reactive protein
